# A meta-analysis of neurogenic exosomes in the diagnosis of Alzheimer's disease

**DOI:** 10.1016/j.heliyon.2023.e20604

**Published:** 2023-10-04

**Authors:** Xin Zhang, Huiyu Liu, Yuanyuan Huang, Ruimin Wang

**Affiliations:** a Neurobiology Institute, School of Public Health, North China University of Science and Technology, Tangshan, Hebei 063210, China; b Dementia and Dyscognitive Key Lab., Tangshan, Hebei 063000, China; c School Basic Medical Sciences, Hebei Key Laboratory for Chronic Diseases, North China University of Science and Technology, Tangshan, Hebei 063210, China

**Keywords:** Alzheimer's disease, Exosome, Blood, Biomarker

## Abstract

**Background:**

Alzheimer's disease (AD) is an irreversible and difficult-to-treat neurodegenerative disease. It is necessary to search for reliable biomarkers for the early diagnosis of AD in a timely and effective manner in high-risk or preclinical AD populations. Studies have shown that neurogenic exosomes in the blood can be effectively used as biomarkers for AD.

**Objective:**

In this meta-analysis, we aimed to find reliable biomarkers (Aβ42, T-tau, and P-tau181 in peripheral blood neurogenic exosomes) for the early diagnosis of AD to provide theoretical support for the early diagnosis of high-risk or preclinical AD populations.

**Methods:**

By searching the literature database, relevant studies on AD diagnostic markers were collected. The study period was from April 1, 2012, to April 1, 2022. The average concentrations of Aβ42, T-tau, and P-tau181 in the exosomes of the AD group and healthy control group were compared using RevMan 5.3 software.

**Results:**

A total of 13 studies were screened, including 842 subjects. Meta-analysis showed that the combined SMD value of neurogenic exosome Aβ42 was 1.70 (95% *CI* = [1.20,2.20], *Z* = 6.69, *P* < 0.05). The combined SMD value of T-tau was 1.02 (95% *CI* = [0.27,1.77], *Z* = 2.67, *P* < 0.05). The combined SMD value of P-tau181 was 1.75 (95% *CI* = [1.16, 2.35], *Z* = 5.75, *P* < 0.05). The levels of neurogenic exosomes Aβ42, T-tau, and P-tau181 in AD patients were significantly higher than those in healthy controls.

**Conclusion:**

Aβ42, T-tau, and P-tau181 in blood neurogenic exosomes can be effectively used as biomarkers for AD and can be applied in the diagnosis, screening, prognosis prediction and disease monitoring of AD.

## 1 Introduction

Alzheimer's disease (AD) is a neurodegenerative disease that is the most common type of senile dementia. Its clinical manifestations are reduced memory and cognitive function, psychiatric symptoms, behavioral disorders, and progressive loss of the ability to live daily lives [1,2]. Its main pathological features are the aggregation and accumulation of Aβ to form senile plaques and excessive phosphorylation of microtubule-related protein Tau to form neurofibrillary tangles. These can lead to neuronal death, synaptic dysfunction, glial cell activation, neuroinflammation and other neurodegeneration [3,4]. As global aging intensifies, the prevalence of AD is on the rise, resulting in a heavy economic and health burden on societies and families. The annual cost of treating AD is currently approximately 1 trillion US dollars, and it is expected to double by 2030 [5,6]. Although there is a great deal of research on AD, there is no effective treatment or cure to date. A handful of five drugs are approved by the U.S. FDA, but these drugs only improve late symptoms or have side effects, and clinical trials for AD dementia or early AD patients have all failed [7,8]. In the AD stage, a large number of nerves in the brain die and are lost, and studies have shown that the pathological changes of AD appear earlier than the clinical symptoms, even more than ten years earlier [[9], [10], [11]]. Therefore, if intervention treatment can be carried out before the occurrence of clinical symptoms of AD, the prevention and treatment effects of AD will be better. Therefore, timely and effective screening and diagnosis of high-risk or preclinical AD populations is particularly important.

The current diagnosis of AD is mainly based on clinical assessment (clinical symptoms and history of disease), neuropsychological scale tests (MMSE, MoCA, ADAS-cog, CDR, and GDS) and neuroimaging (CT, MRI, and PET), as well as biomarker testing (concentrations of cerebrospinal fluid biomarkers Aβ, T-tau, and P-tau). Common AD diagnostic systems include the National Institute of Neurological and Communicative Disorders and the Stroke-Alzheimer's Disease and Related Disorders Association (NINCDS-ADRDA) criteria [[12], [13], [14]], National Institute on Aging-Alzheimer's Association (NIA-AA) criteria [11,15] and International Working Group (IWG) criteria/recommendations [16]. Cerebrospinal fluid biomarkers are the source of AD markers and have been included in the diagnostic criteria for AD [15,17]. However, the detection of CSF markers requires the patient to undergo a lumbar puncture, which is a traumatic procedure that is often difficult for patients to undergo. The neuropsychological scale test is easily affected by the understanding ability and education level of patients, and the test results fluctuate widely. However, neuroimaging examination is difficult to use as a routine screening tool for early AD due to its high detection cost. Therefore, extensive research has focused on finding a more accessible, practical, less invasive and inexpensive AD biomarker that can be applied to widespread screening [[18], [19], [20]]. Blood markers have been promising in the diagnosis, screening, prognostic prediction, and disease surveillance of AD. Moreover, blood collection is minimally invasive, easy to sample, and low cost. With the maturity of detection methods, blood markers have obvious advantages in the implementation in a large number of people.

Exosomes are extracellular vesicles with diameters ranging from 30–100 nm [21]. Exosomes act as a medium of cell communication by transporting contents between cells [22]. Exosomes are secreted by various cell types and can carry relevant proteins, lipids, and nucleic acids, while the lipid bilayer structure of exosomes protects their contents from being degraded by enzymes in the blood [23,24]. Exosomes are found in human body fluids such as blood, saliva and urine, cerebrospinal fluid, and pleural effusion. Because of their small size and ability to cross the blood‒brain barrier (BBB) [25], exosomes can be used as biomarkers to monitor brain-related diseases. Studies have reported that exosomes released by central nervous system cell lines can be detected in peripheral blood [[26], [27], [28]]. Shi et al. injected tau protein labeled with ^125^I into the lateral ventricles of mice with tau gene knockout, and then tau protein labeled with ^125^I was detected in plasma. At the same time, neuron-derived exosomes isolated from plasma were found to carry tau protein labeled with ^125^I [29], indicating that neuron-derived exosomes could be obtained in peripheral blood. Both glial cells and neuronal cell populations release extracellular vesicles that contain carriers of proteins, such as transmembrane proteins, lipids, RNA, and mitochondrial DNA [30]. In addition, exosomes can more easily carry Aβ polypeptides and tau proteins into the blood of the BBB under pathological conditions. Previous studies have also reported that Aβ42, T-tau, and P-tau181 from blood exosomes can accurately diagnose AD and predict its occurrence 10 years before clinical onset. Moreover, the concentrations of Aβ42, T-tau and P-tau181 in peripheral blood neurogenic exosomes can reflect their changes in cerebrospinal fluid [31]. In addition, the number of neurogenic exosomes in blood is significantly correlated with the number of exosomes in brain tissue [32]. These studies suggest that blood neuron-derived exosomes are ideal biomarkers for AD screening in large populations. It has been reported that Aβ42, T-tau, P-tau181, miRNAs, and other proteins in blood neurogenic exosomes can distinguish patients with AD or MCI from healthy individuals [[33], [34], [35], [36], [37], [38], [39], [40]]. However, due to the different detection methods, extraction methods of neurogenic biomarkers and sample sizes, the types of indicators in different studies are uneven, and the results have not been uniformly concluded. We aim to conduct a more comprehensive and systematic review of the published literature and meta-analysis. Relevant data were extracted from the included studies, and Aβ42, T-tau and P-tau181 were used as indicators to obtain effective biomarkers for AD, which can be applied in the diagnosis, screening, prognosis prediction and disease surveillance of AD.

## 2 Methods

### 2.1 Literature search

A literature search was conducted for English or Chinese articles published in PubMed, EMBASE, Web of Science, the Cochrane Library, CNKI and other databases by two researchers. The articles were published between April 1, 2012 and April 1, 2022. For a comprehensive search strategy, relevant articles were retrieved using the following keywords: "Alzheimer's disease", "mild cognitive impairment", "exosomes", "peripheral blood", "neurons", and "biomarkers".

### 2.2 Inclusion and exclusion criteria

The inclusion criteria for the studies were as follows: (1) The diagnosis of AD or MCI was based on the NINCDS-ADRDA or NIA-AA Alzheimer's Criteria; (2) The study included patients with AD or MCI, with healthy individuals serving as controls; (3) The exosomes in this study were exosomes derived from neurogenic cells in blood; (4) The biomarkers Aβ42, T-tau and P-tau181 were evaluated, and quantitative data were provided for evaluation indicators; and (5) The study was published in Chinese or English.

The exclusion criteria were as follows: (1) Reviews, conference abstracts, case reports, or review articles; (2) Repeat articles; (3) Articles that had nothing to do with the evaluation indicators; (4) Articles unrelated to neurogenic exosomes; and (5) Articles that did not include blood samples.

Note Express software was used to summarize the bibliography, duplicate bibliographies were retrieved and deleted, irrelevant bibliographies were removed successively by reading the abstract, the full text of the retained bibliography was downloaded, and the target bibliography was finally obtained according to the inclusion and exclusion criteria.

### 2.3 Data extraction and quality evaluation

Two authors independently screened and selected relevant studies according to the inclusion and exclusion criteria, and then a third author checked them. For studies that did not have specific data in the original text, we attempted to email the author to obtain the original dataset. The extracted data included author name, publication year, author country, sample size, sex, mean age, mental state test (MMSE) score, biomarkers, exosome source, and the isolation and quantitative methods.

After data extraction, the quality of the included studies was evaluated. If there were any differences in the results after verification, we discussed and negotiated to make a joint judgment. The quality evaluation table in ReviewManager5.3 was used to evaluate the included studies. The evaluation was carried out in two parts: bias risk assessment and clinical applicability. A total of 13 questions were evaluated. According to the answers of "yes", "no" or "uncertain" for each question, the corresponding bias risk level was determined as "low", "high" or "uncertain". If the answer to all the signature questions within a range is "yes", the risk of bias can be assessed as low; if the answer to one question is "no", the risk of bias can be assessed as "high".

### 2.4 Statistical analysis

Review Manager 5.3 software was used for statistical analysis of the included literature. Normalized mean difference (SMD) and 95% confidence interval (CI) of evaluation indicators were calculated, and forest maps were generated to compare the mean concentrations of Aβ42, T-tau and P-tau181 of exosomes between the AD group and healthy control group to eliminate the effects of different extraction methods, measurement methods and different dimensions. The χ2 test was used to analyze the heterogeneity among the results, and I^2^ was used to quantify the heterogeneity. If there was no statistical heterogeneity among the results, the fixed effects model was used for meta-analysis. If statistical heterogeneity existed among the results, the random effects model was used for meta-analysis, and subgroup analysis and funnel plot were performed to evaluate the sources of heterogeneity and publication bias in the included studies.

## 3 Results

### 3.1 Literature screening process and quality evaluation results

**Fig. 1:**
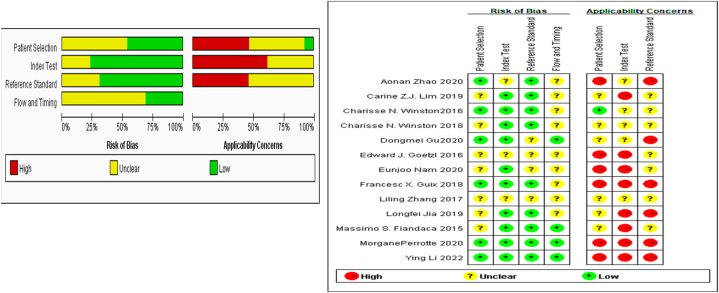
Bar chart and bar chart of bias risk assessment. Different colors (green, red, yellow) and symbols (" + ", "-", "? ") represent "low risk bias," "high risk bias," and "unclear," respectively. The quality assessment chart includes the percentage of bias risk at each level graph, and the level of applicability concerns for each specific item in the study. (For interpretation of the references to color in this figure legend, the reader is referred to the Web version of this article.)

A total of 1925 relevant studies were obtained through initial examination, and 13 studies fully met the inclusion and exclusion criteria of this study. The 13 studies were finally included after layer-by-layer screening [31],[32],[[41], [42], [43], [44], [45], [46], [47], [48], [49], [50], [51]]. The total sample size was 842 cases. All patients with AD or MCI were diagnosed according to the NINCD-ADRDA or NIA-AA criteria, mini-mental status examination (MMSE) score, and clinical histopathological examination. Table 1 shows the general characteristics of the 13 studies included. From the overall level, the risk level of bias in the included studies is relatively low, indicating that the research results are close to the true value. The results of the literature quality evaluation are shown in Fig. 1.

**Table 1:** Characteristics of the eligible studies.

Study	Year	Location	Sample（N）		Age（Mean±SD）		MMSE		Source	Diagnostic criteria	Method	Exosomal Proteins
			AD	Control	AD	Control	AD	Control				
Ying Li	2022	China	76	40	65.8±6.5	65.4±6.4	20.5	29.6	plasma neuron-derived exosomes	NINCDS-ADRDA	NCAM/ABCA1double-labeled	Aβ42, Aβ42/40, Tau, PT181-tau
Aonan Zhao	2020	China	88	80	67.7±4.2	60.3±4.7	17±2.1	29.3±0.7	plasma neuron-derived exosomes	NIA-AA	ExoQuick+L1CAM	Aβ1–42
Eunjoo Nam	2020	Korea	20	26	76.55 ± 1.33	73.92 ± 0.88	16.55 ± 0.52	27.69 ± 0.16	Serum neuron-derived exosomes	NINCDS-ADRDA	ExoQuick EX precipitation solution；CD171/NCAM-L1；Nanoparticle Tracking Analysis	T-tau, p-tau, and amyloid-beta (Aβ42)，
Longfei Jia	2019	China	65	64	65±6	64±5	19.6 ± 3.1	29.3± 1.2	plasma neuron-derived exosomes	NIA-AA	ExoQuick exosome solution+L1CAM+Nanoparticle Tracking Analysis	Ab42, T-tau, and P-T181-tau
Carine Z.J. Lim	2019	Singapore	17	16					plasma neuron-derived exosomes	NIA-AA	APEX platform+Nanoparticle Tracking Analysis	Aβ42
Charisse N. Winston	2018	USA	31	36					plasma neuron-derived exosomes	NINCDS-ADRDA	ExoQuick exosome solution+L1CAM	Aβ1 -42, NRGN,synaptophysin, synaptotagmin, and synaptopodin
Francesc X. Guix	2018	USA	20	10	75.00 ± 11.34	75.90 ± 8.67	15.00 ± 5.24	29.67 ± 0.52	plasma neuron-derived exosomes	NINCDS-ADRDA	ExoQuick exosome solution+L1CAM+Nanoparticle Tracking Analysis	p181 tau，MR tau，FL tau，CD81
Liling Zhang	2017	China	24	14	65.8±5.72	66.4±5.14			plasma neuron-derived exosomes	NIA-AA	ExoQuick exosome solution+L1CAM+Nanoparticle Tracking Analysis	Aβ42, P-T181-tau, P-S396-tau
Edward J. Goetzl	2016	USA	12	10					plasma neuron-derived exosomes	NINCDS-ADRDA	ExoQuick exosome solution	neuro filament light chain, αβ42，BACE-1，λ-Secretase， sAPPα，sAPPβ，Septin-8，GDNF，P-T181-tau，P-S396-tau
Massimo S. Fiandaca	2015	USA	57	57	79.6±6.05	79.6±6.03			plasma neuron-derived exosomes	NINCDS-ADRDA	ExoQuick exosome solution+L1CAM	total tau, P-T181-tau, P-S396-tau, and amyloid b 1–42
Dongmei Gu	2020	China	31	15	68.6 ± 8.0	64.8 ± 6.0	15.9 ± 6.6	27.7 ± 1.7	plasma neuron-derived exosomes	NIA-AA	ExoQuick exosome solution+L1CAM+Nanoparticle Tracking Analysis	Aβ42, p-T181-tau, p-S396-tau, IL-6,MMP-9, CD81
Charisse N. Winston	2016	USA	10	10	75.4 ± 6.8		17.7 ± 0.7		plasma neuron-derived exosomes	NINCDS-ADRDA	ExoQuick exosome solution+L1CAM+Nanoparticle Tracking Analysis	Aβ42, p-T181-tau, p-S396-tau,NRGN, REST
MorganePerrotte	2020	Canada	36	12	79.1 ± 1.1	68.8±1.5	19.9± 1.39	29.42± 0.29	plasma neuron-derived exosomes	NINCDS-ADRDA	Total Exosome Isolation reagent+L1CAM+GFAP	tTau, APP, Aβ42pTau-T181，pTau-T181/tTau，Aβ42/pTau-T181，tTau/Aβ42

### 3.2 Meta-analysis of neurogenic exosomes Aβ42, T-tau and P-tau181

**Fig. 2:**
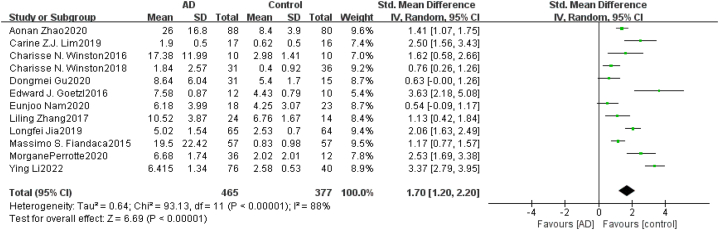
Forest map of the neurogenic exosome Aβ42. The mean level of Aβ42 involved in AD group or control group; total number of AD group or control group; STD Mean Difference, Standardized Mean Difference; CI, confifidence interval; Tau, total heterogeneity; df, degrees of freedom; I^2^, <50% selected the fixed effect model, >50% selected the random effect model.

**Fig. 3:**
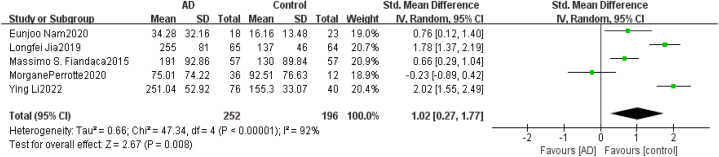
Forest map of neurogenic exosome T-tau. The mean level of T-tau involved in AD group or control group; total number of AD group or control group; STD Mean Difference, Standardized Mean Difference; CI, confifidence interval; Tau, total heterogeneity; df, degrees of freedom; I^2^, <50% selected the fixed effect model, >50% selected the random effect model.

**Fig. 4:**
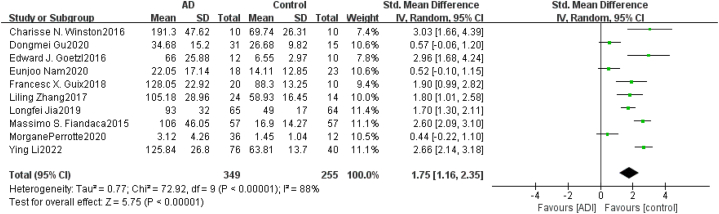
Forest map of neurogenic exosome P-tau181. The mean level of P-tau181 involved in AD group or control group; the number of AD group or control group; STD Mean Difference, Standardized Mean Difference; CI, confifidence interval; Tau, total heterogeneity; df, degrees of freedom; I^2^, <50% selected the fixed effect model, >50% selected the random effect model.

Heterogeneity Test As shown in Fig. 2, for neurogenic exosome Aβ42, the heterogeneity test of the 12 included studies showed that there was a high degree of heterogeneity among the studies (*χ*^*2*^ = 91.13, *P* < 0.05, *I*^*2*^ = 88%). The random effects model was used to combine the effect values, and the combined SMD value was 1.70 (95% *CI* = [1.20,2.20], *Z* = 6.69, *P* < 0.05). The difference between the AD group and healthy control group was statistically significant, indicating that the content of Aβ42 in blood neurogenic exosomes in the AD group was significantly higher than that in the healthy group. For neurogenic exosome T-tau, the heterogeneity test of the 5 included studies showed that there was a high degree of heterogeneity among the studies (χ^2^ = 47.34, *P* < 0.05, *I*^*2*^ = 92%). The random effects model was used to combine the effect values, and the combined SMD value was 1.02 (95% *CI* = [0.27,1.77], *Z* = 2.67, *P* < 0.05), as shown in Fig. 3. The difference between the AD group and the healthy control group was statistically significant, indicating that the content of T-tau in blood neurogenic exosomes in the AD group was significantly higher than that in the healthy group. For neurogenic exosome P-tau181, the heterogeneity test of the 10 included studies showed high heterogeneity among the studies (χ^2^ = 72.92, *P* < 0.05, *I*^*2*^ = 88%). The random effects model was used to combine the effect values, and the combined SMD value was 1.75 (95% *CI* = [1.16, 2.35], *Z* = 5.75, *P* < 0.05), as shown in Fig. 4. The difference between the AD group and the healthy control group was statistically significant, indicating that the content of P-tau181 in blood neurogenic exosomes in the AD group was significantly higher than that in the healthy group.

**Fig. 5:**
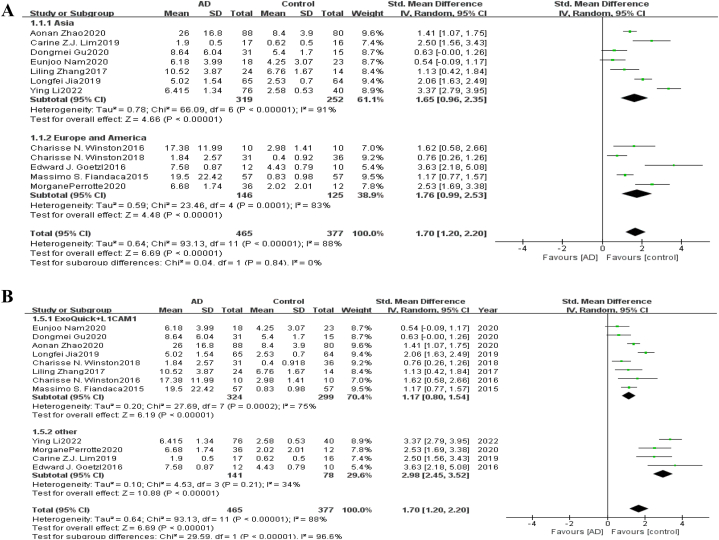
Subgroup analysis of neurogenic exosome Aβ42. (A) is grouped according to Asia, Europe and America; (B) is grouped according to different extraction methods of neurogenic exosomes.

**Fig. 6:**
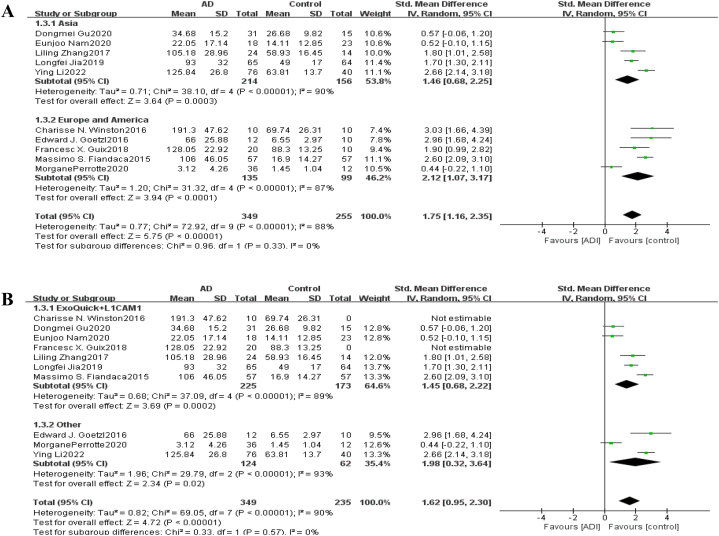
Subgroup analysis of neurogenic exosomes P-tau181. (A) is grouped according to Asia, Europe and America; (B) is grouped according to different extraction methods of neurogenic exosomes.

Subgroup Analysis For neurogenic exosome Aβ42, subgroup analysis was conducted according to regional distribution. A total of 319 AD cases and 252 healthy control cases were included in 7 Asian studies. A total of 146 AD cases and 125 healthy controls were included in 5 studies in Europe and the United States. The results showed that there was still high heterogeneity after regional grouping, and the combined results were statistically significant. This indicates that the regional distribution is not the source of heterogeneity, and there are significant differences in the content of Aβ42 in neurogenic exosomes between AD patients and healthy people in Asia, Europe and the United States. The results of subgroup analysis according to regional distribution are shown in Fig. 5 (A). In addition, subgroup analysis was conducted according to different extraction methods of neurogenic exosomes. A total of 8 studies were conducted using ExoQuick reagent and L1CAM biotin antibody to extract neurogenic exosomes. A total of 324 AD cases and 299 healthy controls were included. A total of 141 AD cases and 78 healthy controls were included in 4 studies with other exosome extraction methods. The results showed that heterogeneity was reduced but still existed after grouping by exosome extraction method, and the combined results were statistically significant. These results indicated that the extraction methods of exosomes were not the main source of heterogeneity, and the two methods had significant differences in the content of Aβ42 in neurogenic exosomes extracted from AD patients and healthy subjects. The results of the subgroup analysis are shown in Fig. 5 (B). For P-tau181, subgroup analysis was also performed according to regional distribution and exosome extraction method, and the results are shown in Fig. 6. The results showed that there was still high heterogeneity after grouping by regional distribution (Fig. 6 A) and exosome extraction methods (Fig. 6 B), and the combined results were statistically significant. These results indicated that the regional distribution and exosome extraction methods were not heterogeneous sources, and the content of P-tau181 in neurogenic exosomes of AD patients and healthy subjects was significantly different.

### 3.3 Sensitivity analysis and publication bias test

A sensitivity analysis was carried out by eliminating individual studies one by one, and the results showed no significant changes, indicating that the results were stable.

**Fig. 7:**
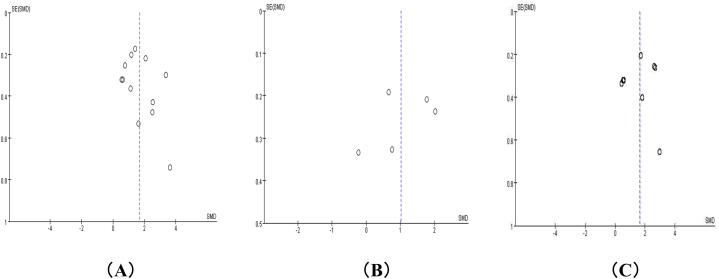
Funnel plot of neurogenic exosome Aβ42, T-tau and P-tau181. (A) is the funnel plot of neurogenic exosome Aβ42; (B) is the funnel plot of neurogenic exosome T-tau; (C) is the funnel plot of neurogenic exosome P-tau181.

A funnel plot was drawn for the publication bias test, and the funnel plot of neurogenic exosome Aβ42 showed that the distribution around each research point was not completely symmetric, suggesting the possible existence of publication bias, as shown in Fig. 7(A). For T-tau and P-tau181, the distribution around each research point is basically symmetrical, suggesting that publication bias may be small, as shown in Fig. 7(B) and (C).

## 4 Discussion

Since the pathogenesis and etiology of AD have not been fully determined, the treatment of AD only slows the progression of the disease, and there is no specific drug or treatment plan for AD at present. The pathological changes of AD are irreversible, so the early stage before the occurrence of the pathological changes of AD and the appearance of clinical symptoms is the key period for the prevention and treatment of AD. Researchers have been searching for reliable, inexpensive, minimally invasive, and easy-to-collect biomarkers to diagnose AD. The goal of our study was to identify an accurate, minimally invasive and easy-to-collect biomarker for Alzheimer's disease. There have been a number of studies on blood markers for Alzheimer's disease, but the results have been inconsistent. By meta-analysis, we combined the results of many such studies to quantitatively and objectively evaluate the differences between these biomarkers in the AD group and the control group. This method can avoid the inconsistency of research results caused by the different selection of research objects and methods, so the possibility of selection bias is small. Meanwhile, the sample size was expanded, and the statistical efficacy of the results was improved.

In our inclusion and exclusion criteria, exosomes were required to be secreted by neurogenic cells. Neurogenic exosomes are isolated by immunoprecipitation using specific antibodies to neuronal protein markers, such as nerve cell adhesion molecules. Since neurogenic exosomes can carry intracerebral neuronal proteins across the blood‒brain barrier into the blood, the protein changes in blood neurogenic exosomes can reflect the changes in intracerebral neuronal proteins. Aβ42, T-tau or P-tau181 in cerebrospinal fluid are currently recognized as the gold standards for the diagnosis of AD [15,17], and studies have shown that the level of exosome biomarkers in AD blood is significantly correlated with that in cerebrospinal fluid [31,52,53]. Therefore, extraction of Aβ42, T-tau or P-tau181 in neurogenic exosomes can more specifically reflect the pathological changes of the central nervous system, suggesting that neurogenic exosome proteins in peripheral blood may be used for clinical diagnosis of AD.

Among the 13 studies included in our analysis, Nam [32]and Perrott [41]et al. believed that neurogenic exosome Aβ42 was not significantly different between the AD group and control group, while Perrott [41] believed that neurogenic exosome T-tau was not significantly different between the AD group and control group. Nam [32], Perrott [41] and Guix [47] believed that neurogenic exosome P-tau181 was not significantly different between the AD group and the control group, while other selected studies showed that Aβ42, T-tau and P-tau181 were significantly different between the AD group and the control group. Aβ42, T-tau and P-tau181 are suitable as biomarkers for the diagnosis of AD. However, through a comprehensive meta-analysis of these 13 studies, we found that the levels of neurogenic exosome Aβ42, T-tau and P-tau181 in AD patients were significantly higher than those in healthy controls. The regional distribution of different studies and different methods of exosome extraction and identification did not have a great impact on the combined index. The results indicate that Aβ42, T-tau and P-tau181 in blood neurogenic exosomes can be effectively used as biomarkers for AD, which can be applied in the diagnosis, screening, prognosis prediction and disease monitoring of AD. We expanded the sample size of the AD group and the control group through meta-analysis, which can avoid the problem of inconsistent results caused by small sample sizes and different experimental methods in a single study.

Exosomes are particles secreted by a variety of cell types [54], so exosomes can carry AD-related proteins such as Aβ, and they can be transmitted through synapses throughout the brain [55]. Given their small size and membrane-like structure, exosomes can easily cross the blood‒brain barrier [56]. It has been reported that intravenously injected exosomes can cross the blood‒brain barrier and deliver substances to brain cells, triggering specific changes [57]. The blood‒brain barrier is pathologically damaged in AD brains [58], so exosomes can easily transport Aβ and tau proteins to the blood through the blood‒brain barrier. In addition, the circulation of cerebrospinal fluid also causes exosomes to spread from the central nervous system to the peripheral blood, thus carrying out "disease transmission". Therefore, the changes in blood exosome Aβ42, T-tau and P-tau18 can reflect the pathological changes in the brain caused by AD.

Previous studies have shown that Aβ42 in blood is not a biomarker for AD [59], which is inconsistent with our conclusion. After analyzing the reasons, Aβ in blood is not only from the central nervous system but also a small amount of Aβ is from platelets [60], while tau has a short half-life and is easily degraded in blood, thus causing inaccurate research results. In the study we selected, the indicators were Aβ42, T-tau and P-tau181 transported by the exosomes secreted by nerve cells to the peripheral blood. The results of the exosome bilayer lipid membrane could effectively avoid the contents from blood dilution or enzymatic degradation. Meanwhile, CD81 was used for the standardization of exosomes, and the difference caused by the content of exosomes in the samples was eliminated. Whether neurogenic exosome proteins in blood can truly be biomarkers for the diagnosis of AD still needs much clinical practice. If they can be effectively used as biomarkers of AD, on the one hand, they will contribute to the clinical diagnosis of AD and reduce the economic pressure and physical harm of patients, and at the same time, early diagnosis can significantly improve the prevention and treatment effect of AD. On the other hand, neurogenic exosome proteins can be used to accurately detect neuronal injury in the brain, which can provide targets and new drug delivery vectors for AD treatment.

However, this study also has some limitations. First, this study can distinguish AD patients from healthy subjects and cannot be divided into AD, MCI or preclinical AD. Therefore, longitudinal comparisons can be made according to the disease development process, which needs further research. Second, it has been found that in the scheme of separating neuronal exosomes from blood, thrombin D used may be mixed with the tau protein in rabbit brain, thus affecting the experimental results. However, most current research schemes use thrombin D, and recombinant thrombin can effectively improve the accuracy of the results. Therefore, it is worthy of attention in future research.

## 5 Conclusions

In our study, we found that Aβ42, T-tau and P-tau181 in blood neurogenic exosomes can effectively distinguish the AD population from the healthy population. Although further clinical trials are needed to verify whether blood exosomes can be used as biomarkers for the screening and diagnosis of AD, the results of our meta-analysis are suitable.

## Author contribution statement

Xin Zhang; Ruimin Wang: Conceived and designed the experiments; Performed the experiments; Analyzed and interpreted the data; Contributed reagents, materials, analysis tools or data; Wrote the paper. </p>

Huiyu Liu; Yuanyuan Huang: Conceived and designed the experiments; Analyzed and interpreted the data; Contributed reagents, materials, analysis tools or data; Wrote the paper. </p>

## Declaration of competing interest

The authors declare that they have no known competing financial interests or personal relationships that could have appeared to influence the work reported in this paper.
